# Nursing care management or nursing case management? A scoping review to identify models, roles, and advantages

**DOI:** 10.1097/nmg.0000000000000368

**Published:** 2026-05-29

**Authors:** Piergiorgio Martella, Nicola Rossi, Elisa Schiavoni, Daniele Napolitano

**Affiliations:** **Piergiorgio Martella**, Neuroscience Department, Neurosurgery Care Management Fondazione Policlinico Gemelli IRCCS in Rome, Italy; **Nicola Rossi**, Internal Medicine, Dell'Angelo Hospital, ULSS3 in Mestre, Italy. **Elisa Schiavoni**, CEMAD, Fondazione Policlinico Gemelli IRCCS in Rome, Italy; and **Daniele Napolitano**, SITRA - Scientific Direction, Fondazione Policlinico Gemelli IRCCS in Rome, Italy.

**Keywords:** care coordination, case management models, continuity of care, nursing care management, nursing case management, patient-centered care

## Abstract

**Background:**

Care coordination is increasingly essential for addressing complex health needs across inpatient and outpatient settings. However, nursing care management and nursing case management are frequently used interchangeably, resulting in conceptual ambiguity and challenges in comparing outcomes across studies.

**Purpose:**

To map and synthesize the literature on nursing care management and nursing case management; clarify professional roles and core functions; and summarize associated clinical, organizational, and economic outcomes in adult care pathways.

**Methods:**

A scoping review was conducted following Joanna Briggs Institute methodology and PRISMA-ScR guidelines. Searches across multiple databases identified studies involving adult inpatients and outpatients receiving nursing care or case management interventions. Data from 29 included studies were charted and synthesized narratively.

**Results:**

Both roles shared key components, including assessment, care planning, education, coordination, and follow-up. Interventions were associated with improved self-management, quality of life, patient experience, and continuity of care, as well as reduced emergency department use and hospital readmissions. Evidence predominantly focused on nursing case management, with fewer studies explicitly addressing nursing care management.

**Conclusions:**

Despite demonstrated benefits, persistent variability in definitions, role delineation, outcome measures, and economic evaluation limits comparability. Greater standardization of roles, caseloads, governance, outcome indicators, and cost assessment is needed to strengthen implementation, evaluation, and scalability of nursing care and case management models.

**Figure:**
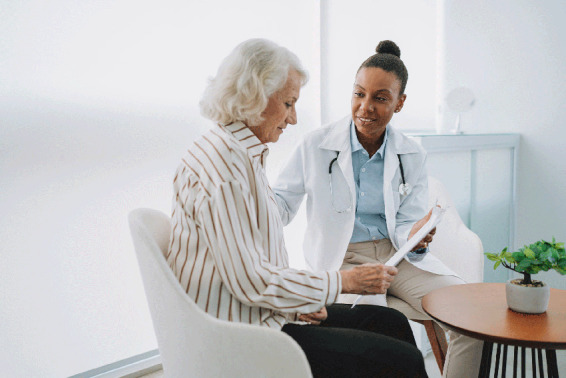
No caption available.

In recent decades, the increasing complexity of health care needs, population aging, and the rising prevalence of chronic diseases have profoundly transformed health care delivery models.^1^,^2^ Within this evolving landscape, the roles of nurse care manager and nurse case manager have gained increasing recognition as a strategic element for the comprehensive management of patients and the coordination of care pathways across hospital and community settings.^3^ Nursing care management and nursing case management are conceptually related approaches that share many goals, although they're not entirely synonymous.^4^ However, in both clinical and organizational practice, the terms “nursing care manager” and “nursing case manager” are often used interchangeably, leading to terminological ambiguity and difficulties in comparing findings across studies.^5^

The concept of nursing case management originated in the US in the 1970s, initially within the social and community care sectors, to coordinate services and resources for frail and complex patients.^6^ Over time, this model evolved into a clinical and multidimensional approach that integrates assessment, planning, implementation, and evaluation of care.^7^ Nursing care management represents a broader and more proactive evolution of this concept, emphasizing continuity of care, patient empowerment, and the promotion of self-management and therapeutic education.^8^

Although both models share the common aim of ensuring quality, continuity, and efficiency of care, they differ in scope and level of intervention: nursing case management focuses on individualized, case-specific care coordination, whereas nursing care management operates at a systematic or population level, addressing groups of patients with chronic or complex conditions.^9^,^10^

Both the nurse care manager and the nurse case manager have advanced competencies in multidimensional assessment, individualized care planning, coordination across care settings, and monitoring of clinical and organizational outcomes.^10^,^11^ These professionals serve as liaisons between patients and their families, facilitating communication among health care providers and ensuring seamless service integration. They play a strategic role in both hospital settings—by managing complex discharges, preventing readmissions, and reducing length of stay—and in community settings, where they contribute to chronic care management and disease-specific care pathways.^12^

The international literature identifies several organizational models for implementing nursing care management and nursing case management, shaped by the health care system, level of integration, and service organization.^13^ In Italy, these management models have been progressively integrated into the national framework through the National Chronic Care Plan, where the nurse care manager is formally recognized as a key figure for achieving the goals of integrated care, in line with international literature on patient-centered care.^14^,^15^ A substantial body of evidence supports the positive impact of nursing care management and nursing case management on clinical, organizational, and experiential outcomes.^16^,^17^ For example, at the clinical level, nursing care management interventions are associated with improved treatment adherence, better symptom control, and reduced exacerbation among chronic patients; whereas at the organizational level, the presence of a nursing case manager contributes to reduced hospital length of stay, fewer readmissions, and more efficient use of resources.^15^,^18^ From the experiential perspective, patients and families report greater satisfaction, clearer communication, and a stronger sense of continuity of care.^19^

However, the literature also highlights persistent variability and challenges. The lack of a shared definition of the role, differences in nurse training, and heterogeneity of outcome indicators hinder comparability among studies and limit the standardization of evaluation methods.^20^ Based on these considerations, the present article aims to identify and analyze the existing literature on nursing care management and nursing case management, describe these professional roles and their key functions, summarize the primary clinical and organizational outcomes, and outline the current state of knowledge on these essential figures in health care. Ultimately, the goal is to provide an integrated and up-to-date overview that supports the dissemination of evidence-based, patient-centered care models and value-based care models and strengthens the contribution of nursing care and case management to health care system sustainability.

## METHODS

### Review methodology

This scoping review was conducted in accordance with the methodological guidance of the Joanna Briggs Institute (JBI), and the PRISMA Extension for Scoping Reviews (PRISMA-ScR).^21^,^22^ The review adhered to its preregistered protocol, available on the Open Science Framework under the DOI 10.17605/OSF.IO/4CGXD and accessible at the following link: https://archive.org/details/osf-registrations-4cgxd-v1.

This scoping review aimed to explore what is currently known about intra-hospital care and case management of adult inpatients and outpatients; to describe the roles and functions of the care and case manager; and to identify the principal clinical, organizational, and health care outcomes associated with the implementation of care/case management models. Consistent with the PCC (Population/Participants, Concept, Context) framework, the review focused on adult patients as the target population, examined care and case management models and the professional role of the care/case manager as the central concept, and considered inpatient and outpatient health care settings as the contextual boundaries of the analysis.

### Search strategy

Facet analysis was used to identify all keywords and MeSH terms consistent with the research method. The following keywords were identified: nurs∗ care management, nurs∗ case management, nurse care manager, nurse case manager, hospital, inpatient, outpatient, intervention, model, strategy, strategies, and continuity of care. Truncation (∗) was used to retrieve word variants.

In addition, the following MeSH terms were identified: Case management, Inpatients, and Outpatients. The search string was constructed for each database consulted, using the previously selected terms. The strings were run on each database on April 1, 2025.

### Inclusion and exclusion criteria

The inclusion criteria for this scoping review were studies involving adult patients, with no restrictions on study design (for example, quantitative, qualitative, or mixed methods). Only articles published in Italian or English and reporting evidence related to inpatient or outpatient settings were included. Studies involving pediatric populations, addressing primary health care settings, or published in languages other than Italian or English were excluded.

### Data charting process

In accordance with the JBI methodology for scoping reviews, the data charting process was conceived as an iterative and interactive procedure. Two reviewers (SE and RN) independently developed a standardized data-charting form based on the objectives of the review and the PCC framework identified in the protocol. The form was first piloted using a small sample of included studies to ensure the clarity, consistency, and relevance of the extracted variables. Any discrepancies or ambiguities emerging during this phase were resolved by two expert supervisors (MP and ND), and the form was subsequently refined.

The final data-charting framework included descriptive information on study characteristics (authors, year, country, setting, study design, sample); definitional aspects of care and/or case management; details of the intervention (core components, professional roles, organizational model); and reported clinical, organizational, and economic outcomes.

Throughout the process, two reviewers conducted data charting independently to ensure reliability, and supervisors resolved any conflicts. The charting process remained flexible, allowing the addition of new variables emerging from the included studies, in line with JBI recommendations.

### Data extraction

Data extraction was performed using the final version of the charting tool and applied consistently to all articles included in the review. Two reviewers (SE and RN) independently extracted data. Extracted domains reflected the structure of *Supplementary Table 1*, http://links.lww.com/NMT/A13 (such as author/year; setting; study design/sample; model definition; main activities performed; outcomes at clinical, organizational, and economic levels; and specific notes or gaps), ensuring alignment with the preestablished conceptual framework of the review.

The use of Rayyan facilitated screening and identification of the final sample, but data extraction itself was conducted manually to allow in-depth appraisal of key constructs.^23^ Any discrepancies in the extracted information, particularly regarding definitions, intervention components, and outcomes, were reviewed by the two expert supervisors. When needed, additional verification was carried out by reconsulting the full text. No automation tools were used for extraction to preserve interpretative accuracy and maintain adherence to JBI's emphasis on manual, reviewer-driven charting.

### Data synthesis

Given the heterogeneity in study designs, populations, intervention types, and outcome measures, a narrative descriptive synthesis approach was adopted. Synthesis proceeded in three structured phases: description of the mapping of evidence, categorization of outcomes (clinical, organizational, and economic), and identification of gaps and research needs. The results of the synthesis were presented narratively and supported by detailed tables to ensure transparency and facilitate comparison across studies.

## RESULTS

**FIGURE 1:**
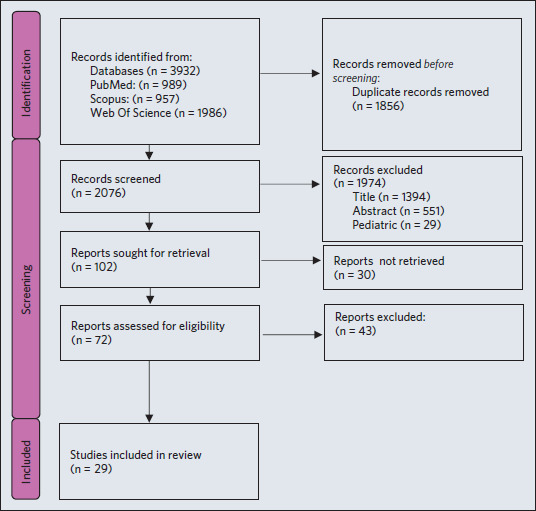
PRISMA-ScR flow diagram

The literature search retrieved 3932 papers, and after duplicates were removed, 2076 remained. Overall, 72 articles were considered potentially eligible. After full-text assessment, 43 articles were excluded, and 29 were included. The PRISMA-ScR flow diagram shows the flow chart of articles through the scoping review (see Figure 1). The results of the scoping review are detailed in *Supplementary Table 1*, http://links.lww.com/NMT/A13.

### Knowledge about care/case management

The included studies describe care and case management interventions across diverse hospital settings, including emergency departments (EDs), renal transplantation, oncology, multimorbidity and chronic disease management, psychiatric care, rheumatoid arthritis, and frail older adult patients.^24^-^34^ Interventions were reported in both inpatient and outpatient settings, often with structured follow-up to ensure continuity of care.^35^,^36^ Several studies also examined nursing care management during transition phases, such as hospital discharge to community services.^37^,^38^

### Definition of care and case management

Multiple studies explicitly referred to the definition provided by the Case Management Society of America, describing case management as a collaborative process of assessment, planning, facilitation, coordination, evaluation, and advocacy to meet an individual's health needs.^24^,^36^,^39^ Other frameworks cited include the German Society of Care and Case Management (DGCC) and the Swiss Network Case Management.^27^,^28^

Mororó and colleagues proposed a definition of nursing care management as the coordination and integration of managerial and care actions through leadership and interaction with health professionals and patients.^40^ In several articles, no formal definition was reported; case management was instead described through its practical activities, such as individualized planning, coordination of resources, and patient navigation.^25^,^41^

### The role of the nursing care manager and the nursing case manager

Most studies identified the nurse case manager as the central figure.^25^,^29^,^31^,^34^ In several contexts, nurses were supported by multidisciplinary teams, including physicians, pharmacists, psychologists, and social workers.^7^,^27^,^41^ Nursing case and nursing care managers take different roles—such as coordinator of discharge processes, provider of patient education and counseling, facilitator of screening and linkage to care, supporters of psychosocial needs—and have essential attributes such as decision-making and cooperative relationships.^28^,^33^,^34^,^38^,^40^,^42^,^43^ Advanced practice nurses were highlighted as case managers addressing both clinical and social determinants of health, particularly in US hospitals.^44^

### Clinical, organizational, and health care/economic outcomes

***Clinical outcomes***. Several studies reported and improved adherence to therapy and self-management, reduction in ED utilization among frequent users, and enhanced quality of life.^24^,^25^,^27^,^34^,^39^,^42^,^45^ Specific clinical benefits included reduced disease activity in patients with rheumatoid arthritis and reduced depressive symptoms, lower suicide risk, and increased cancer screening uptake among patients with psychiatric disorders.^32^,^34^,^46^

***Organizational outcomes***. Case management interventions were frequently associated with improved continuity of care and better coordination across hospital and community settings.^31^,^37^ Reduced hospital readmission or unplanned acute care use was observed in multiple studies.^25^,^35^ Enhanced communication within teams and with patients was also reported.^28^,^47^

***Health care and economic outcomes***. Economic results were heterogeneous. Some studies demonstrated reductions in health care costs linked to fewer hospitalizations and shorter stays, whereas others found no significant cost savings at the hospital or payer level.^25^,^27^,^45^ The cost-effectiveness of nurse-led case management models has been reported in advanced heart failure.^39^ In specific contexts, increased out-of-pocket expenses for preventive treatments were observed.^42^

## DISCUSSION

**FIGURE 2:**
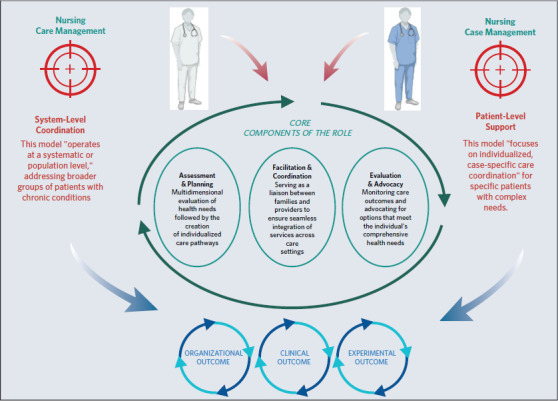
Core components of the nursing care manager and nursing case manager roles

The aim of this scoping review was to analyze the professional roles of nursing care managers and nursing case managers, distinguishing between them and describing their core activities (see Figure 2). The literature frequently uses the terms interchangeably, but the two roles can be conceptually distinguished according to various aspects, summarized in Table 1.

**TABLE 1:** Conceptual distinction between nursing care management and nursing case management

Dimension	Nursing care management	Nursing case management
**Scope**	Population-oriented care coordination	Individual-patient–focused coordination
**Focus**	Chronic disease management and care pathways	Management of complex individual cases
**Level of intervention**	System or program level	Patient-specific level
**Aim**	Continuity and proactive management of patient populations	Coordination of services for a specific patient
**Typical activities**	Monitoring populations, care pathway coordination, prevention	Individual care planning, navigation, discharge coordination

A first relevant finding concerns the number of studies identified: among the 29 articles included, only three specifically addressed the figure of the nursing care manager. A possible explanation for this finding lies in the historical and conceptual evolution of the two roles. Case management has been more extensively studied in the international literature, particularly in North American health care systems, where it has long been used as an organizational strategy to coordinate care for complex patients. Consequently, this term has become the dominant terminology in research and policy documents. In contrast, the concept of nursing care management is often embedded within broader chronic care or integrated care models and may therefore be described using different terms, without explicitly referring to the role of the nursing care manager.

Additionally, the substantial conceptual overlap between nursing care management and nursing case management may lead authors to use the two terms interchangeably, further limiting the explicit identification of nursing care management as a distinct research focus. This represents a critical gap and suggests that, although the two roles are theoretically distinct, the literature has predominantly focused on the case manager. This imbalance reflects both a conceptual and operational overlap between the two roles, which often share common goals and intervention strategies.

However, the two professional profiles are clearly differentiated in their formal definitions. The nursing care manager is responsible for ensuring the continuity of care for a specific patient population.^8^ In contrast, the nursing case manager guarantees care continuity for an individual patient.^6^ However, the analysis of the included studies reveals that, in practice, these roles frequently intersect. According to Mororó and colleagues, nursing care management involves “the coordination and integration of care and management actions through leadership, interaction, communication, and cooperative relationships among nurses, health care professionals, and service users.”^40^ Conversely, case management is described as “a collaborative process of assessment, planning, facilitation, care coordination, evaluation, and advocacy for options and services to meet an individual's and family's comprehensive health needs through communication and available resources to promote quality and cost-effective outcomes.”^24^

Both roles share the overarching goal of ensuring care continuity and quality by coordinating professionals and services.^30^,^31^ These are relatively new professional figures whose adoption across medical fields is steadily increasing, thus attracting growing research interest. Despite this, substantial variability persists in the activities these professionals perform across care settings.^36^,^37^,^42^ This flexibility may reflect the evolving nature of nursing care management and nursing case management roles, which often adapt to the specific needs of patient populations and health care systems. However, the lack of standardized role definitions and intervention components may limit comparability across studies and make it difficult to consistently evaluate the impact of these models on clinical and organizational outcomes.

The studies included in this review, though primarily addressing the case manager, highlight that both roles can be implemented flexibly across a range of health care contexts, ensuring that patients receive the most appropriate care along their clinical pathways.^24^,^27^,^31^ Both roles have demonstrated effectiveness in inpatient and community settings, promoting continuity of care and management of potential complications.^35^,^36^

Although the functions and objectives of both figures are recognized, significant variability remains in how their activities are operationalized and their roles defined.^25^,^33^ Most studies addressing the case manager adopt the conceptual framework proposed by the Centers for Medicare & Medicaid Services or equivalent models (such as DGCC or the Swiss Network Case Management). Others describe the concept mainly through practical activities rather than within a clear theoretical framework.^27^ This heterogeneity indicates that a standardized operational model remains lacking and that professionals tend to adapt their practice to the needs of the patient population and care context.

In the reviewed literature, both care and case managers are depicted as responsible for developing and coordinating patient care pathways. However, some studies not included in this review conceptualize case management as a process implemented by multidisciplinary teams rather than a single designated professional.^47^

Given the current demographic trend of population aging, the need for patient management outside of hospital settings has increased. This shift requires professionals capable of ensuring continuity and coordination of care across settings.^7^ The nurse case manager plays a key role in bridging hospital and community care, supporting patient education, promoting self-management, and advocating for personalized care.^34^,^42^

Evidence suggests that these professionals improve clinical outcomes by enhancing treatment adherence, disease awareness, and self-care capabilities.^37^,^43^ Improvements in quality of life and psychosocial well-being are consistently reported, confirming that case management not only affects clinical indicators but also fosters patient empowerment and trust. Nevertheless, several large randomized controlled trials have reported limited or nonsignificant effects, suggesting that the effectiveness of case management may depend on the intensity of the intervention, patient engagement, and contextual factors such as organizational support and digital integration.^48^

To ensure care continuity, nurse care managers typically serve as core members of multidisciplinary teams, collaborating closely with other health care professionals.^49^ The integration of this role within team-based care models has been shown to improve communication, enhance workflow efficiency, and facilitate patient transitions between the hospital with its internal services and community settings.^7^,^27^,^41^,^50^ A reduction in acute episodes, complications, and ED visits has also been observed, although these outcomes appear to vary depending on intervention type, health care system structure, and therapeutic focus.^25^,^35^ These findings suggest that the effectiveness of nursing care management and nursing case management interventions is strongly dependent on context. Factors such as intervention design, health care system organization, and the degree of integration between hospital and community services appear to influence the outcomes observed across studies. This highlights the importance of considering organizational and health system contexts when designing and evaluating nursing care management and nursing case management models.

Economic and managerial outcomes also show considerable variability. Some studies report potential cost savings from reduced hospital admissions and shorter length of stay, whereas others find no significant financial benefits for health care organizations or systems.^27^,^45^ Such discrepancies likely arise from differences in intervention duration, cost assessment methods, and whether community care expenses are included. Nevertheless, cost-effectiveness analyses, such as those concerning nurse-led heart failure management, suggest that structured case management models may yield long-term value by reducing avoidable hospital use and improving chronic disease outcomes. However, long-term studies assessing the sustainability and cost-effectiveness of these roles remain scarce. Extended monitoring of these professionals' activities would enable more accurate evaluations, but such analyses are currently limited by the relatively recent and heterogeneous implementation of these roles.

### Limits and future prospects

This review has several methodological limitations. First, the existing literature is unbalanced: the majority of studies focus on nursing case management, whereas nursing care management remains underrepresented. This imbalance limits the possibility of directly comparing the two roles and may lead to conclusions that primarily reflect the characteristics of case management models. Furthermore, operational definitions are heterogeneous and often not grounded in shared theoretical frameworks; this conceptual variability makes it difficult to compare interventions and may affect the consistency of outcome interpretation across studies.

The included studies exhibit marked heterogeneity in several domains, including the health care contexts (such as EDs, oncology, psychiatry, chronic disease management), the types of outcomes assessed (clinical, organizational, and economic), and the indicators used to measure the impact of nursing care management and nursing case management interventions. This variability complicates direct comparisons across studies and limits the identification of the most effective intervention components.

Another limitation concerns the limited information regarding the educational pathways required to qualify as a nurse care manager or a nurse case manager. This gap may reflect incomplete reporting within the included studies, but it may also indicate a genuine variability in professional preparation across health care systems, where training requirements and role formalization differ substantially.

Methodologically, most studies employ descriptive or qualitative designs without control groups or standardized measures, with limited transparency regarding sampling criteria and analytical procedures. Few studies evaluate the effectiveness or economic sustainability of care and case management models. Overall, the field remains exploratory and requires more robust, longitudinal, and theoretically grounded research to consolidate evidence and clarify the professional boundaries of both roles.

Given these findings, future research should prioritize the distinction between nursing care management and nursing case management; explore their differential impact across clinical contexts; and assess their long-term effectiveness and cost-effectiveness to inform policies, workforce plannings, and organizational decision-making.

## IMPLICATIONS FOR NURSE LEADERS

The findings of this review highlight how the variability in role definitions, intervention components, and outcome indicators currently limit the comparability and evaluation of nursing care management and nursing case management models. In this context, nurse leaders have a critical role in reducing conceptual and operational variability between nursing care management and nursing case management to enable more consistent implementation, evaluation, and scalability. Leaders should adopt a shared local taxonomy and translate it into clear job descriptions, competency requirements, and accountable workflows across settings. Although initial standardization may occur at the organizational level, alignment with national- or system-level frameworks is essential to support comparability across institutions and facilitate the broader implementation of nursing care management and nursing case management models.

Operational parameters, such as eligibility criteria, referral pathways, caseload standards, and escalation rules, should be explicitly defined and embedded within multidisciplinary governance and information-sharing processes. Finally, nurse leaders should establish a standardized evaluation framework that links the core components of these models (assessment, individualized care planning, education/support, coordination, and follow-up) to a common set of outcomes spanning patient experience and self-management, clinical indicators, unplanned service use (such as emergency visits/readmissions), and economic measures that include community and postdischarge resource utilization to support benchmarking and evidence-informed decisions on sustainability and scale-up.

## CLARIFICATION AND STANDARDIZATION

This scoping review outlines the current landscape of nursing care management and nursing case management across hospital and outpatient settings, showing substantial overlap in their practical application despite conceptual distinctions. Evidence is predominantly centered on nursing case management, whereas studies explicitly addressing nursing care management remain limited, highlighting a clear gap in the literature. Overall, these models support improvements in clinical outcomes, continuity of care, and patient engagement, with documented benefits in treatment adherence, symptom monitoring, quality of life, and organizational coordination. However, economic findings remain inconsistent across studies, likely reflecting differences in health care system structures, intervention intensity, and cost evaluation methods.

This variability, together with the heterogeneity in role definitions and intervention components, limits the comparability of existing evidence and complicates the evaluation of nursing care management and nursing case management models across contexts. Strengthening role clarity and developing more standardized operational frameworks may therefore represent a crucial step for advancing both research and practice. Clear definitions, shared outcome indicators, and consistent role descriptions would support more robust evaluation of these models and facilitate their integration into patient-centered and value-based health care systems.
